# Residential Proximity to Major Roadways and Risk of Type 2 Diabetes Mellitus: A Meta-Analysis

**DOI:** 10.3390/ijerph14010003

**Published:** 2016-12-22

**Authors:** Zhiqing Zhao, Faying Lin, Bennett Wang, Yihai Cao, Xu Hou, Yangang Wang

**Affiliations:** 1 Emergency Department, Maternal and Children Health’s Hospital of Tangshan, Tangshan 063000, China; zystsfy@yeah.net; 2 Department of Endocrinology, Affiliated Hospital of Qingdao University, Qingdao 266003, China; bennettzhwang@gmail.com; 3 Department of Medical Services, The Eighth Hospital of PLA, Shigatse 857000, China; hongyandl@yeah.net; 4 Department of Microbiology, Tumor and Cell Biology, Karolinska Institute, Stockholm 17177, Sweden; caoyhsw@126.com

**Keywords:** type 2 diabetes, residential proximity to major roadways, meta-analysis

## Abstract

Research indicates that higher levels of traffic-related pollution exposure increase the risk of diabetes, but the association between road proximity and diabetes risk remains unclear. To assess and quantify the association between residential proximity to major roadways and type 2 diabetes, a systematic review and meta-analysis was performed. Embase, Medline, and Web of Science were searched for eligible studies. Using a random-effects meta-analysis, the summary relative risks (RRs) were calculated. Bayesian meta-analysis was also performed. Eight studies (6 cohort and 2 cross-sectional) with 158,576 participants were finally included. The summary unadjusted RR for type 2 diabetes associated with residential proximity to major roadways was 1.24 (95% confidence interval [CI]: 1.07–1.44, *p* = 0.001, I^2^ = 48.1%). The summary adjusted RR of type 2 diabetes associated with residential proximity to major roadways was 1.12 (95% CI: 1.03–1.22, *p* = 0.01, I^2^ = 17.9%). After excluding two cross-sectional studies, the summary results suggested that residential proximity to major roadways could increase type 2 diabetes risk (Adjusted RR = 1.13; 95% CI: 1.02–1.27, *p* = 0.025, I^2^ = 36.6%). Bayesian meta-analysis showed that the unadjusted RR and adjusted RR of type 2 diabetes associated with residential proximity to major roadways were 1.22 (95% credibility interval: 1.06–1.55) and 1.13 (95% credibility interval: 1.01–1.31), respectively. The meta-analysis suggested that residential proximity to major roadways could significantly increase risk of type 2 diabetes, and it is an independent risk factor of type 2 diabetes. More well-designed studies are needed to further strengthen the evidence.

## 1. Introduction

The epidemic of type 2 diabetes has increased in both, developed and developing countries, and has become a serious health issue worldwide [1,2]. There is also strong evidence that type 2 diabetes can increase risks of cardiovascular disease, stroke, cancer and other diseases [2,3,4,5]. Previous studies have suggested that the epidemic of type 2 diabetes is in large part attributable to obesity and the growing aging population in contemporary society [3]. Other factors causing the increasing prevalence of type 2 diabetes have also been suggested, such as smoking and lifestyle factors [6,7,8]. However, those established risk factors cannot explain all the increasing prevalence of type 2 diabetes.

Recent studies have focused on the causal role of air pollution in the development of type 2 diabetes, and have found that air pollution increases risk of type 2 diabetes [9,10,11,12,13,14,15]. Individuals residing near major roadways will be exposed to higher levels of traffic related pollutants compared with those residing far away from major roadways [16,17]. Though previous studies have shown that higher levels of traffic-related pollutants can increase risk of type 2 diabetes [9,10,11,12,13,14,15], no studies have focused solely on the association between residential proximity to major roadways and type 2 diabetes risk. Several studies investigating the association between air pollutants and type 2 diabetes also reported data on the association between residential proximity to major roadways and type 2 diabetes, but the findings were contradictory [10,11,12,13,18,19]. Therefore, in contrast to the evidence for the adverse impact of air pollutants on type 2 diabetes, there is lack of evidence for the casual association between road proximity and type 2 diabetes. 

There is an important need to develop a better understanding of the association between road proximity and type 2 diabetes. In this meta-analysis, we presented a quantitative assessment of the evidence from studies reporting data on the association between residential proximity to major roadways and type 2 diabetes. The present meta-analysis was registered at PROSPERO (CRD42014009214).

## 2. Materials and Methods

### 2.1. Literature Search and Inclusion Criteria

Embase, Medline, and Web of Science were searched to identify eligible studies. The following key words were used: (residential proximity to major roads OR proximity to major roads OR distance to road OR distance to roadway OR residential distance OR traffic pollution OR traffic main road OR major roads) and (diabetes OR diabetic). Additional articles were identified by reviewing the reference lists of relevant studies. There was no language restriction. The literature search was performed on 20 September 2016.

The following inclusion criteria were used in the meta-analysis: (1) Cohort or cross-sectional studies; (2) Estimating the association between residential proximity to major roadways and type 2 diabetes; (3) Type 2 diabetes was diagnosed by World Health Organization criteria or criteria recommended by countrywide guidelines; (4) Reported relative risks (RR) or other risk estimates for type 2 diabetes. Studies without usable data or those with overlapping data were excluded.

### 2.2. Data Extraction and Quality Assessment

For each included study, the following data were extracted: name of the first author, date of publication, study location, exposure type, time of follow-up, number of participants, events of type 2 diabetes, adjusted factors, and RRs with 95% Confidence Interval (CI). Both unadjusted and adjusted risk estimates were extracted, and we would perform meta-analyses on unadjusted and adjusted risk estimates separately. The quality assessment was performed by Newcastle Ottawa scale (NOS) [20]. Methodological quality was mainly assessed on the selection of participants, the comparability of exposure group and non-exposure group, and the ascertainment of outcomes. Studies with 7–9 stars were defined to have A-level quality, those with 4–6 stars were defined to have B-level quality, and those with 0–3 stars were defined to have C-level quality.

### 2.3. Statistical Analysis

The I^2^ statistic was used to estimate the heterogeneity, and I^2^ > 50% indicated high degree of heterogeneity among included studies [21]. The summary RRs with 95% CI were calculated using a random-effects meta-analysis [22]. We performed meta-analyses on unadjusted and adjusted estimates separately. Subgroup analysis were performed by study design (Cohort or Cross-sectional) and the definitions of residential proximity to major roadways. In the sensitivity analysis, the influence of single study on the summary RRs was observed by omitting that corresponding study. We also performed a sensitivity analysis through Bayesian meta-analysis, which had the advantage of naturally allowing for full uncertainty and could capture the substantial heterogeneity and the variability from all sources [23,24]. Further sensitivity analysis was performed by excluding those studies in which roadway proximity was based on self-report data or how it was defined was not provided. A normal distribution (0, 10^7^) was used for coefficient parameters, and an inverse gamma distribution (0.001, 0.001) was for the variance. The summary statistics for parameters were calculated after 200,000 iterations (50,000 for burn-in). Publication bias was judged by funnel plot and Egger’s test [25]. Bayesian meta-analysis was performed by use of WinBUGS software (version 1.4.2, MRC Biostatistics Unit, Cambridge Biomedical Campus, Cambridge, UK). Other statistical analyses were done with Stata 12.0 (StataCorp LP, College Station, TX, USA). *p*-value less than 0.05 was statistically significant.

## 3. Results

### 3.1. Study Selection and Characteristics

**Figure 1:**
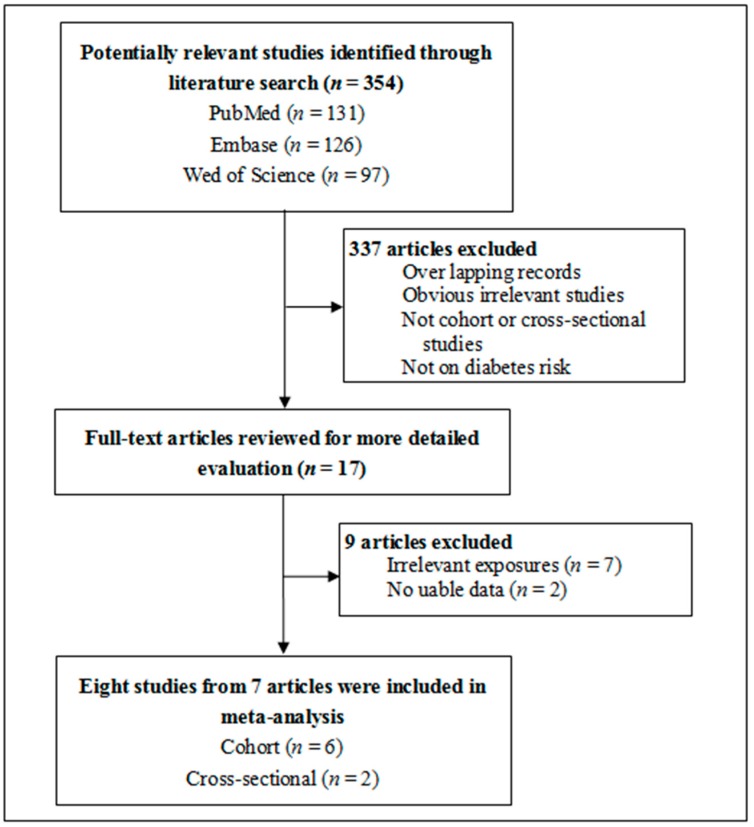
Flow chart of study selection in this meta-analysis.

Of 354 identified articles, eight studies from seven articles with 158,576 participants were finally included into the meta-analysis (Figure 1) [9,10,11,12,13,18,19]. The study by Puett et al. reported two different cohort studies [11]. There were a total of 7657 cases of type 2 diabetes. Table 1 shows the main characteristics of those eight studies (Table 1). There were six cohort studies [9,11,12,18,19] and two cross-sectional studies [10,13]. Two studies were from USA [11], three studies were from Germany [9,18,19], and the other three studies were from Denmark [12], Bulgaria [13], and Netherlands [10], respectively. Five studies defined residential proximity to major roadways as less than 100 m to major roadways, two studies defined it as residential houses located near to roads with high traffic intensity, and one study defined it as less than 50 m to major roadways (Table 1). The definitions of major roadways used in those included studies were different (Table 1). There were two studies using data reported by participants to define the high traffic intensity, five studies using the residential address and the public traffic data to define major roadways, and one study in which how major roadway was defined was not reported (Table 1). The adjustment factors used in those included studies were also different (Table 1). According to the NOS criteria, five studies [11,12,18,19] had A-level quality and the other three studies [9,10,13] had B-level quality (Table 1).

### 3.2. Meta-Analysis

**Figure 2:**
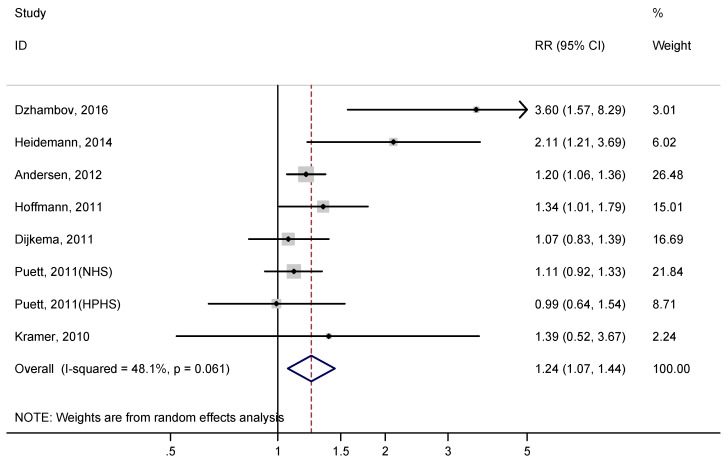
Unadjusted relative risk (RR) of type 2 diabetes associated with residential proximity to major roadways.

Heterogeneity in the meta-analysis of unadjusted risk estimates was high (I^2^ = 48.1%). The summary unadjusted RR for type 2 diabetes associated with residential proximity to major roadways was 1.24 (95% CI: 1.07–1.44, *p* = 0.001) (Figure 2). 

**Figure 3:**
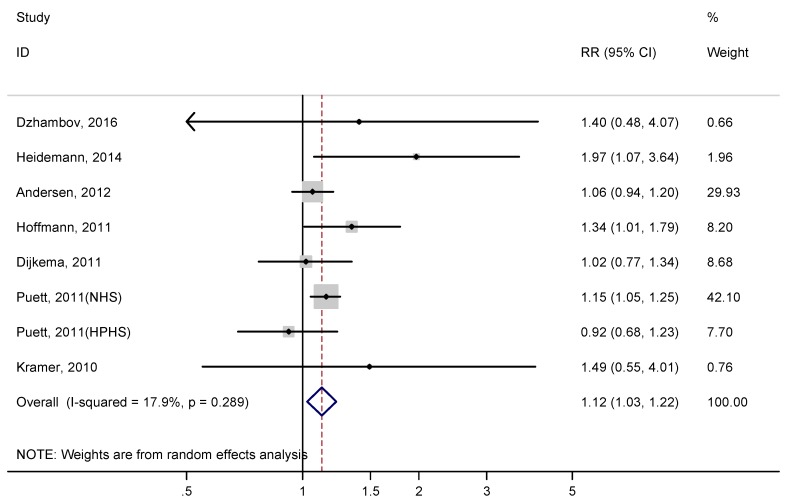
Adjusted relative risk (RR) of type 2 diabetes associated with residential proximity to major roadways.

Heterogeneity in the meta-analysis of adjusted risk estimates was low (I^2^ = 17.9%). The summary adjusted RR of type 2 diabetes associated with residential proximity to major roadways was 1.12 (95% CI: 1.03–1.22, *p* = 0.01) (Figure 3). Sensitivity analysis found that no single study had an obvious influence on the summary RRs. Subgroup analysis using data from 6 cohort studies further revealed that residential proximity to major roadways could increase type 2 diabetes risk (Adjusted RR = 1.13; 95% CI: 1.02–1.27, *p* = 0.025, I^2^ = 36.6%). However, meta-analysis of those two cross-sectional studies didn’t find an association between residential proximity to major roadways and type 2 diabetes risk (RR = 1.04; 95% CI: 0.80–1.36, *p* = 0.771, I^2^ = 0%).

There were six studies examining residential proximity to major roadways and two studies examining residential proximity to roads with high traffic intensity (Table 1). After excluding those two studies examining residential proximity to roads with high traffic intensity, meta-analysis of the remaining six studies showed that residential proximity to major roadways could still significantly increase type 2 diabetes risk (RR = 1.11, 95% CI: 1.04–1.19, *p* = 0.001, I^2^ = 1.3%). Meta-analysis of those two studies examining residential proximity to roads with high traffic intensity also found that residential proximity to roads with high traffic intensity significantly increased risk of type 2 diabetes (RR = 1.81, 95% CI: 1.06–3.08, *p* = 0.028, I^2^ = 0%). After excluding those studies in which roadway proximity was based on self-report data or how it was defined was not provided, meta-analysis of five studies using the residential address and the public traffic data to define major roadways still showed that residential proximity to major roadways could increase type 2 diabetes risk (Adjusted RR = 1.10; 95% CI: 1.03–1.18, *p* = 0.004, I^2^ = 0%).

Bayesian meta-analysis showed the unadjusted RR and adjusted RR of type 2 diabetes associated with residential proximity to major roadways were 1.22 (95% CI: 1.06–1.55) and 1.13 (95% CI: 1.01–1.31), respectively. The outcomes from Bayesian meta-analysis provided further evidence for the association between residential proximity to major roadways and type 2 diabetes risk.

**Figure 4:**
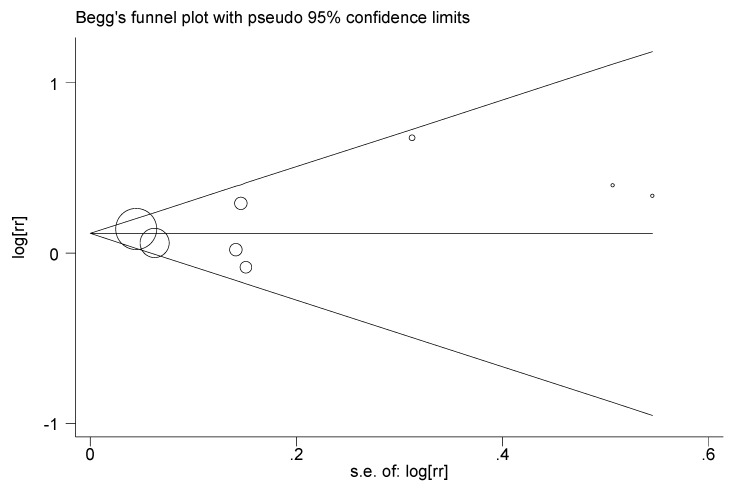
Funnel plot for assessing publication bias risk in the meta-analysis.

Publication bias was not evident in the funnel plot of this meta-analysis (Figure 4), and the *p* value from Egger’s test was 0.46.

## 4. Discussion

The meta-analysis provided a quantitative assessment of the evidence from studies assessing the association between residential proximity to major roadways and type 2 diabetes. It’s the first meta-analysis on this topic and has great reference value for future studies. Eight individual studies with 158,576 participants were finally included, which would provide a reliable quantitative assessment. The findings from the meta-analysis suggested residential proximity to major roadways significantly increased type 2 diabetes risk (Unadjusted RR = 1.24, 95% CI 1.07–1.44, *p* = 0.001). After adjusting for possible confounding factors, residential proximity to major roadways independently increased type 2 diabetes risk (Adjusted RR = 1.12, 95% CI 1.03–1.22, *p* = 0.01). Therefore, the meta-analysis suggested that residential proximity to major roadways could significantly increase risk of type 2 diabetes, and it’s an independent risk factor of type 2 diabetes.

Heterogeneity in the meta-analysis of adjusted risk estimates was low (I^2^ = 17.9%), which suggested the consistency of the influence of residential distance to main road on type 2 diabetes. In addition, sensitivity analysis found that no single study had obvious influence on the summary RRs, which proved the credibility of the summary risk estimates. Finally, the outcomes from Bayesian meta-analysis provided further evidence for the association between residential proximity to major roadways and type 2 diabetes risk. Thus, the meta-analysis provided strong evidence for the association between residential proximity to major roadways and type 2 diabetes.

There are several possible explanations for the association between residential proximity to major roadways and type 2 diabetes. Firstly, NO_2_, PM_2.5_ and PM_10_ are main pollutants of traffic-related air pollution, and previous studies have suggested that road proximity is significantly correlated with higher concentrations of particulate matters and NO_2_ [26,27,28]. Previous studies have shown that higher levels of air pollutants, such as NO_2_, PM_2.5_ and PM_10_, can lead to insulin resistance, which may further result in the development of diabetes [12,29,30]. Some studies have directly suggested that higher levels of NO_2_, PM_2.5_ and PM_10_ can lead to increased risk of diabetes and diabetes-related mortality [31,32,33]. Individuals residing near major roadways will be exposed to higher levels of traffic-related pollutants compared with those residing far away from major roadways, and thus may have higher risk of type 2 diabetes; Secondly, previous studies have demonstrated that traffic-related air pollutants can cause adverse health effects, especially inflammation-related effects [34,35,36]. The role of inflammation in the pathogenesis of type 2 diabetes has been well established [37,38]. Because traffic-related air pollutants can cause inflammation and oxidative stress in human bodies, individuals residing near major roadways will be exposed to higher levels of traffic-related air pollutants, and thus have high-grade inflammation and may further suffer from increased risk of type 2 diabetes; Thirdly, previous studies also suggest that traffic noise is associated with diabetes risk [13,39]. Individuals residing near major roadways are exposed to higher level of traffic noise, and thus may suffer from increased risk of diabetes; Finally, obesity is a major cause of the epidemic of type 2 diabetes [1,2]. A recent study suggested that residential proximity to major roadways was associated with higher overall and abdominal obesity, which provided another explanation for the association between residential proximity to major roadways and type 2 diabetes [40].

The impact of residential proximity to major roadways on human heath has gained more and more attentions in recent years [41,42,43,44,45,46]. Recent studies suggest that residential proximity to major roadways can result in increased risks of many diseases, such as cardiovascular disease, cancer and hypertension [41,42,43,44,46,47,48,49]. The present meta-analysis suggests that residential proximity to major roadways significantly increases risk of type 2 diabetes, and itis an independent risk factor of type 2 diabetes, which further adds new evidence for the adverse impact of residential proximity to major roadways on human health. The findings from our study also suggest possible approaches for the prevention of type 2 diabetes. In addition, considering the adverse impact of residential proximity to major roadways on human heath, city planning should be carefully designed to reduce its adverse impact [50].

There were several limitations in the meta-analysis. Firstly, a major limitation of the meta-analysis was the inherent biases from observational studies. Both cohort studies and cross-section studies are unable to eliminate the risk of inherent biases caused by residual confounding factors. Some factors were not considered in the exposure assessment of those included studies. There are several other factors needing to be considered to improve the validity of methods used for exposure assessment, such as the method for geocoding addresses, types of traffic, number of years at the residence, types of buildings included or predominating, types of windows and the number of hours at home or away. Future studies considering these factors above in the exposure assessment are needed. Secondly, there were obvious differences in the exposure characterization approach and adjustment factors used in those included studies. Those differences could cause the high-degree heterogeneity across included studies, and could not be ignored when interpreting the pooled results in the meta-analysis. Though both random-effect meta-analysis and Bayesian meta-analysis found a significant association between residential proximity to major roadways and type 2 diabetes risk, the finding of this meta-analysis still needs to be validated by more studies in the future. Thirdly, cohort studies are usually better than cross-sectional studies in exploring risk factors of diseases. However, there were limited numbers of studies assessing the association between residential proximity to major roadways and type 2 diabetes, and thus both cross-sectional studies and cohort studies were considered eligible in the meta-analysis. More cohort studies are needed to further strengthen the evidence. Fourthly, there was risk of information bias caused by the low reliability of self-report data in some included studies. Two included studies used self-report data to define the high traffic intensity, and the reliability of these two reports may be low. However, a sensitivity analysis of five studies using the residential address and the public traffic data to define major roadways still found a significant impact of residential proximity to major roadways on type 2 diabetes risk. Finally, there was a lack of relevant studies from developing countries or non-white population. All those included studies were performed in developed countries. Additional studies from non-white population or developing countries are needed to assess the impact of residential proximity to major roadways on type 2 diabetes risk. 

## 5. Conclusions

In summary, the meta-analysis suggested that residential proximity to major roadways significantly increased risk of type 2 diabetes, and it’s an independent risk factor of type 2 diabetes. More well-designed studies are needed to further strengthen the evidence. In addition, further studies from non-white population or developing countries are also needed.

## Figures and Tables

**Table 1:** Characteristics of seven included studies in the meta-analysis.

Study	Baseline Study Dates	Country	Study Design	Follow-Up Time	Participants	Number of Events	Residential Distance to Major Roadways	Definitions of Major Roadways or High Traffic Intensity	Adjustment Factors	Quality *
Dzhambov, 2016 [13]	2014	Bulgaria	Cross-sectional	NA	513	35	Home located near to roads with high traffic intensity	Extreme traffic intensity reported by participants.	Sex, age, socioeconomic classes, occupations, dietary habits, alcohol consumption, PM_2.5_, loud noise, and smoking.	B
Heidemann, 2014 [19]	1997–1998	Germany	Cohort	12.1 years	3604	252	Home located near to roads with high traffic intensity	Extremely busy traffic reported by participants.	Sex, age, smoking, heating of house, educational status, BMI, waist circumference, sport activity, and parental history of diabetes.	A
Andersen, 2012 [12]	1993–1997	Denmark	Cohort	9.7 years	51,818	2877	<50 m from major roadways	A road with at least 10,000 vehicles/day which was determined by the residential address and the public traffic data.	Adjusted for sex, hypertension, hypercholesterolemia, myocardial infarction, BMI, waist-to-hip ratio, smoking status, smoking duration, smoking intensity, environmental tobacco smoke, educational level, physical/sports activity in leisure time, alcohol consumption, fruit consumption, fat consumption, and calendar year.	A
Hoffmann, 2011	2000–2003	Germany	Cohort	5 years	3398	309	<100 m from major roadways	A road with busy traffic but how it was defined in details was unclear.	Adjusted for sex, age, body mass index, education, smoking, physical activity, and city of residence.	B
Dijkema, 2011 [10]	1998–2000	Netherlands	Cross-sectional	NA	8018	213	<100 m from major roadways	A road with at least 5000 vehicles/day which was determined by the residential address and the traffic data from Geographical Information System.	Adjusted for average monthly income, age (continuous) and gender.	B
Puett, 2011 NHS [11]	1989	USA	Cohort	13 years	74,412	3784	<100 m from major roadways	Major roadways, such as interstates highways and major noninterstate roads which was determined by the residential addresses and the public traffic data.	Adjusted for age, season, calendar year, state of residence, time-varying cigarette smoking (status and pack-years), time-varying hypertension, baseline BMI, time-varying alcohol intake, baseline physical activity, and time-varying diet.	A
Puett, 2011 HPHS [11]	1989	USA	Cohort	13 years	15,048	688	<100 m from major roadways	Major roadways, such as interstates highways and major noninterstate roads which was determined by the residential addresses and the public traffic data.	Adjusted for age, season, calendar year, state of residence, time-varying cigarette smoking (status and pack-years), time-varying hypertension, baseline BMI, time-varying alcohol intake, baseline physical activity, and time-varying diet.	A
Kramer, 2010 [18]	1985–1994	Germany	Cohort	16 years	1775	187	<100 m from major roadways	A road with more than 10,000 cars/day which was determined by the residential addresses and data on road traffic from environmental agency.	Adjusted for age, BMI, heating with fossil fuels, workplace exposure with dust/fumes, extreme temperatures, smoking, and education.	A

* Quality was assigned as A quality with 7–9 stars, B quality with 4–6 stars, and C quality with 0–3 stars; USA = United States of America; BMI, body mass index; NHS, Nurses’ Health Study; HPFS, Health Professionals Follow-Up Study; NA, not available.
